# Vaccinia virus growth factor induces divergent signaling programs and biological outcomes from cellular epidermal growth factor

**DOI:** 10.1371/journal.ppat.1014725

**Published:** 2026-10-26

**Authors:** Anil Pant, Lara Dsouza, Zhilong Yang

**Affiliations:** Department of Veterinary Pathobiology, College of Veterinary Medicine & Biomedical Sciences, Texas A&M University, College Station, Texas, United States of America; University of Utah, UNITED STATES OF AMERICA

## Abstract

Epidermal growth factor receptor (EGFR) signaling is a major pathway that controls cellular growth, survival, proliferation, and differentiation. Viruses often hijack this pathway to create a favorable environment to support their replication. Vaccinia virus (the prototype member of *poxviridae*) encodes its own viral growth factor (VGF). VGF is a secreted viral polypeptide with sequence and structural homology to the cellular EGF, which binds to and activates EGFR signaling. Studies from our lab and others have shown that VGF promotes metabolic reprogramming, cell motility, virulence, and spread during VACV infection. However, it remains unclear whether VGF fully mimics EGF signaling or selectively rewires downstream pathways to meet the specific demands of viral replication. To address this question, we used quantitative global phosphoproteomics to systematically compare signaling programs induced by EGF and VGF in primary human foreskin fibroblasts. By analyzing global phosphorylation patterns, kinase activity inference, and motif enrichment, we found that although both peptides engage the same EGFR receptor, EGF and VGF induce distinct downstream phosphorylation and bias downstream kinase activities. Transcriptomic analysis provided a complementary view of the cellular responses to the ligand treatment. Moreover, we found that VGF is more effective at promoting VACV plaque formation than EGF, thereby providing functional support to the VGF-biased EGFR signaling nodes important for viral spread. These findings highlight differences in how viral and cellular growth factors activate EGFR signaling and position poxviral growth factors as powerful tools for examining the fundamental principles of cellular signaling.

## Author summary

Viruses often manipulate cell signaling pathways to create environments that promote viral replication and spread. One critical pathway is EGFR signaling, which controls how cells grow, move, and survive. Vaccinia virus, the prototype member of the poxvirus family, produces its own mimic of cellular epidermal growth factor called VGF. Although VGF resembles the EGF peptide, it is unclear whether it activates cells in the same way or rewires the pathway to benefit the virus. We compared the response of human primary fibroblasts to EGF and VGF using quantitative global phosphoproteomics. We found that although both ligands engage the same receptor, EGFR, they trigger distinct patterns of protein phosphorylation and downstream kinase engagement. RNA sequencing further identified complementary transcriptional changes that were consistent with the ligand-specific pathway differences observed in the phosphoproteomic analysis. Functionally, we found that these differences enable VGF to be more effective than EGF at promoting vaccinia virus spread. Our work reveals how a virus‑encoded growth factor reshapes a major cellular pathway and highlights poxviruses as valuable tools for understanding the basic principles of cell signaling.

## Introduction

Epidermal growth factor receptor (EGFR) signaling pathway is a central regulator of cell proliferation, metabolism, and survival. Upon ligand binding, EGFR dimerizes and autophosphorylates, leading to the activation of a cascade of downstream kinases that coordinate metabolic activity and cell-cycle progression [1–3]. EGFR signaling is important for enabling cells to respond to growth cues and environmental stress. It is also essential for tissue repair and epithelial homeostasis in organs such as the skin, lungs, and gastrointestinal tract. EGFR dysregulation is associated with inflammatory diseases and is a major driver of various cancers [2,4,5]. Because EGFR integrates growth, metabolic, and transcriptional programs, it becomes an attractive target for hijacking by viruses that must reprogram host cells for their replication and spread. By co-opting EGFR signaling, viruses may create a cellular environment that favors viral gene expression, metabolic rewiring, and support efficient viral replication and spreading.

Poxviruses exemplify this strategy by encoding a viral growth factor. Vaccinia virus (VACV, the prototype member of the *poxviridae* family) encodes two copies of the viral growth factor (VGF) gene, C11R, within the inverted terminal repeats of its genome. VGF is a secreted viral polypeptide with sequence and structural homology to cellular epidermal growth factor (EGF) and transforming growth factor [6–9]. VGF shares approximately 50% sequence similarity with EGF, but also has distinct biochemical features, which include glycosylation that is absent in EGF. VGF is the most highly expressed of the VACV early genes [10,11], and previous studies have shown its important role in VACV replication and virulence in resting cells and *in vivo* [12,13]. Consistent with these roles, VGF induces mitogenic signaling [14,15], promotes cell motility, and enhances viral spread [8,16–18], processes that are energetically demanding and likely require coordinated regulation of host metabolism and signaling [19]. In support of this, we previously showed that VACV reprograms cellular metabolism, including the tricarboxylic acid cycle, neutral lipid levels, and pyrimidines, in a VGF-dependent manner in infected cells [20–22]. We further showed that a recombinant peptide corresponding to the processed, secreted variant of VGF rescues the reduction in EGFR phosphorylation observed in cells infected with the VGF-deleted virus, supporting VGF’s role as a major activator of EGFR signaling during VACV infection. Although these findings support the long-standing view that VGF is a poxviral homolog of cellular EGF, they also raise an important question: to what extent does VGF recapitulate the spectrum of EGFR signaling, or instead selectively rewires downstream pathways to meet the specific needs of viral replication?

In this study, we answer this question by combining quantitative phosphoproteomics and transcriptomics to systematically compare EGFR signaling in primary human fibroblasts treated with cellular EGF and vaccinia virus VGF peptides. Because VGF is a peptide secreted early during infection, with autocrine and paracrine effects, this approach mimics physiological conditions and examines how uninfected neighboring cells may be affected by VGF to support VACV spread. By analyzing global phosphorylation patterns, kinase activity inference, and motif enrichment, we found that EGF and VGF induce distinct patterns of phosphosite utilization, resulting in differences in downstream kinase activation despite engaging the same receptor. RNA sequencing further supported the distinct cellular responses identified by phosphoproteomics. Our data show that EGF biases EGFR signaling towards the canonical ERK-RSK and PI3K-Akt-mTOR pathways associated with sustained anabolic growth, whereas VGF biases signaling toward selective branches, including Rho/Rac and stress-associated MAPKs such as p38 and JNK. We found that VGF treatment enhances plaque formation in the VGF-deleted VACV (vΔVGF) compared to EGF, indicating that ligand-biased EGFR signaling has functional consequences that promote more organized spread. Together, these findings reveal fundamental differences in how viral and cellular growth factors engage EGFR signaling and provide insights into how poxviruses adapt host signaling networks to support viral replication.

## Results

### Design and overview of phosphoproteomic analysis

We previously expressed, purified, and characterized a peptide corresponding to the cleaved, secreted form of VGF expressed in mammalian cells, demonstrating its ability to induce EGFR phosphorylation [20,22]. Importantly, treatment with this VGF peptide during infection with a VGF deletion mutant virus (vΔVGF) restored EGFR phosphorylation to levels comparable to WT-VACV infection, whereas EGF failed to fully rescue EGFR activation under the same experimental conditions [20]. Moreover, stimulation of uninfected cells with EGF or VGF peptides induced distinct effects on key proteins associated with mTOR signaling, pyrimidine metabolism, and lipid metabolism [20,22]. These observations suggest that the recombinant VGF peptide is biologically active and functionally distinct from EGF, providing a strong rationale to define ligand-specific signaling differences downstream of EGFR.

We performed quantitative phosphoproteomics analysis in uninfected primary human foreskin fibroblasts (HFFs) treated with vehicle, EGF, or VGF with four biologically independent replicates per treatment (Fig 1A). This allows us to directly compare how each ligand engages EGFR and downstream signaling networks without the confounding effects of other viral factors or infection-induced stress responses. Because VGF is secreted, studying its effects in uninfected cells provides insight into how VACV alters signaling in neighboring cells and promotes viral spread. HFFs were used in this study because they have unaltered signaling and metabolic pathways compared with transformed cell lines, making them well-suited for examining growth factor-driven responses. Our previous studies also highlight that HFFs provide a robust system for studying VGF-dependent metabolic and signaling changes during VACV infection [20–23]. To minimize background signaling from serum-derived factors, we serum-starved HFFs before VGF or EGF treatment.

**Fig 1:**
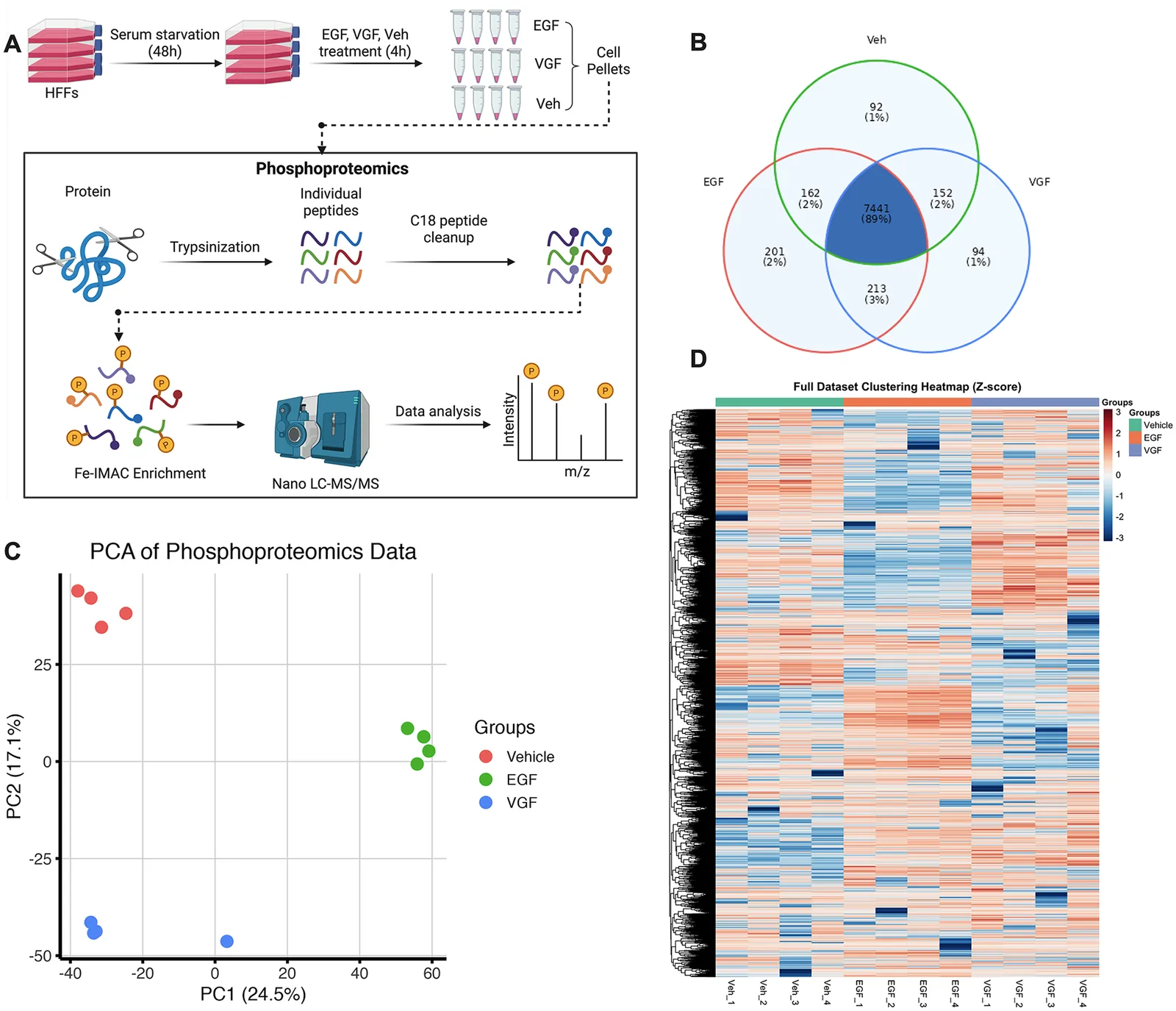
Phosphoproteomic profiling of HFFs treated with vehicle, EGF, or VGF. **(A)** A simplified experimental workflow for phosphoproteomics. HFFs were serum starved for 48 h followed by treatment with vehicle (PBS), EGF (5μg/mL), or VGF (5μg/mL) for 4 h. Cell pellets were collected, proteins were digested into peptides, phosphopeptides were enriched using Fe-IMAC, and samples were analyzed by nano LC-MS/MS. The phosphoproteomics experiments were performed with four independent biological replicates (n = 4) per treatment group. **(B)** Venn diagram showing the overlap of quantified phosphosites across vehicle, EGF, and VGF treatment groups. **(C)** Principal component analysis (PCA) of phosphosite intensities showing separation of samples based on treatment conditions. **(D)** Heatmap of row-wise Z-score-normalized phosphosite intensities. Phosphosites were hierarchically clustered, whereas sample columns were displayed in their predefined experimental order by treatment group. Fig 1A Created in BioRender. Pant, A. (2026) https://BioRender.com/9fc0fqg.

We identified more than 8,350 reproducibly quantified phosphosites across vehicle, EGF, and VGF-treated conditions (Fig 1B). The majority of phosphosites were shared among vehicle, VGF, and EGF treatments, reflecting basal phosphorylation. A substantially distinct subset of phosphosites showed ligand-specific regulation between EGF and VGF, indicating divergent phosphorylation responses. Biological replicates within each treatment group clustered tightly in the sample correlation matrix with high Pearson correlation coefficients (S1A Fig), indicating strong reproducibility across samples. Sample-to-sample variability was also low across the same treatments, with median coefficients of variation of approximately 16% for EGF, 20% for vehicle, and 23% for VGF (S1B Fig). Log_2_-transformed phosphosite intensity distributions were comparable across treatments (S1C Fig), and density plots showed consistent alignment of signal distributions across samples following normalization (S1D Fig). Overall, these results confirm the robustness of our data and indicate that differences observed in downstream analyses were not driven by technical artifacts. Principal component analysis (PCA) revealed a clear separation of EGF-, VGF-, and vehicle-treated samples, with tight clustering of biological replicates (Fig 1C), indicating robust and treatment-specific signaling responses. Consistent with this, hierarchical clustering of regulated phosphosites showed distinct phosphorylation patterns for each treatment, with EGF and VGF inducing partially overlapping yet clearly separable signaling profiles (Fig 1D). These results show that although VGF and EGF activate many of the same EGFR signaling pathways, they also produce distinct patterns of phosphorylation, suggesting that viral and cellular ligands propagate EGFR signals in different ways.

### EGF and VGF induce distinct phosphorylation patterns

We next performed phosphosite-level differential analyses across treatment conditions. Volcano plots show that both VGF and EGF induce robust, partially overlapping phosphorylation relative to the vehicle (S2A Fig, S2B Fig). VGF treatment resulted in fewer phosphorylation changes than the EGF treatment, compared to the vehicle treatment. Direct comparison of EGF and VGF peptide treatments (Fig 2A) identified numerous phosphosites that differed between EGF and VGF stimulation. Representative phosphosites with established relevance to the signaling pathways discussed in this study are labeled in the volcano plots to indicate the magnitude of representative phosphorylation changes. In the direct comparison between EGF and VGF peptide treatments (Fig 2B), the most differentially regulated sites occurred on serine residues, with fewer on threonine and very few on tyrosine. A similar pattern was observed in the EGF versus vehicle and VGF versus vehicle comparisons (S2C Fig, S2D Fig). Across all conditions, serine phosphorylation accounted for the majority of regulated sites, which may partly reflect the higher abundance of serine phosphorylation in eukaryotic cells and its greater detectability on global phosphoproteomics profiling platforms [24,25]. While serine residues represented most regulated phosphosites, ligand-specific differences were observed in both serine- and threonine-centered phosphorylation patterns.

**Fig 2:**
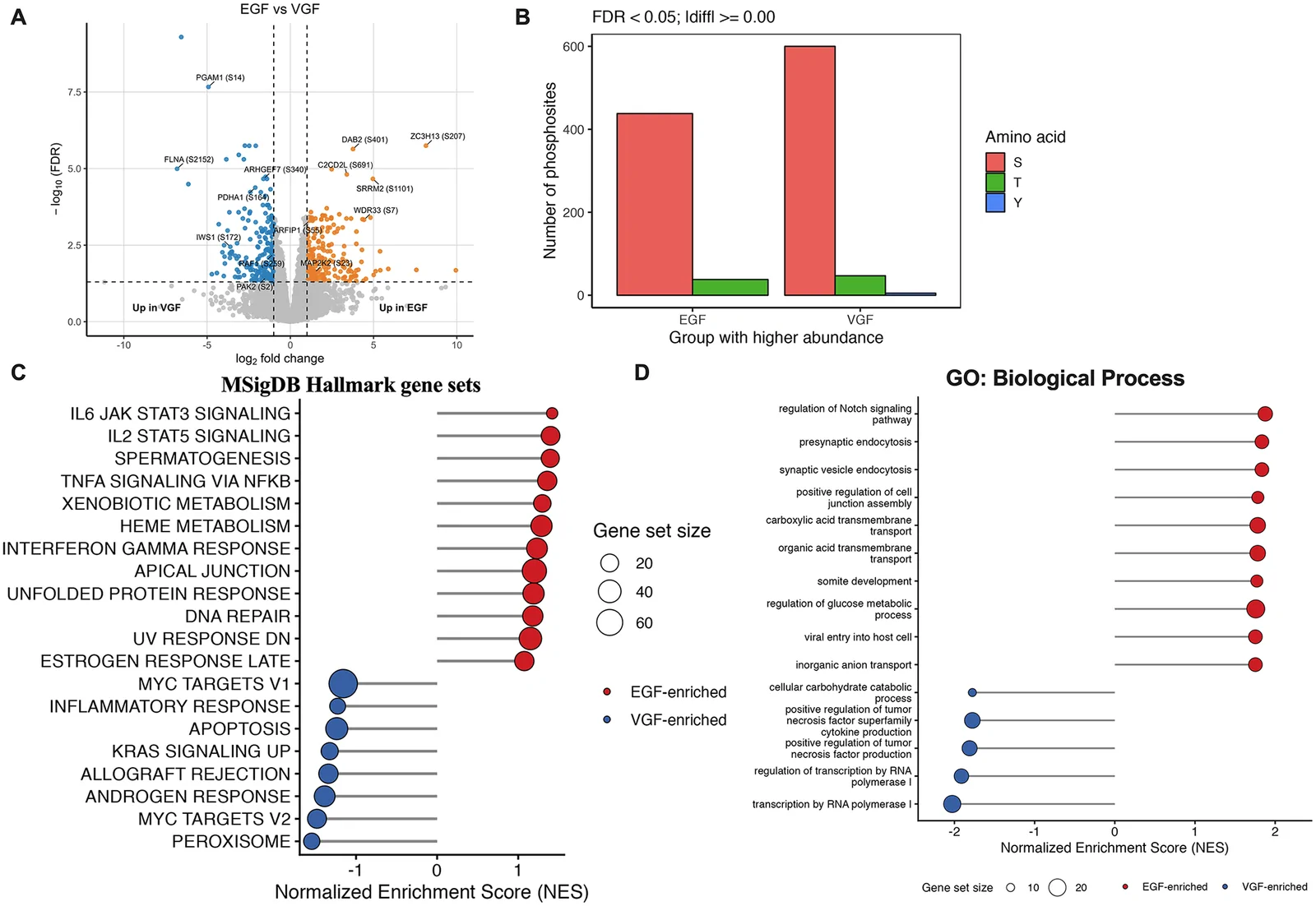
Differential phosphosite and pathway enrichment analyses distinguish EGF- and VGF-driven signaling. **(A)** Volcano plot showing differential phosphosite abundance between EGF- and VGF-treated HFFs, plotted as log_2_ fold change versus −log_10_(FDR), with significantly regulated phosphosites defined by FDR < 0.05 and |log_2_ fold change| ≥ 1. Representative phosphosites relevant to the signaling pathways discussed in this study are labeled. **(B)** Bar plot summarizing significantly regulated phosphosites (FDR < 0.05) grouped by amino acid residue (serine, threonine, tyrosine) and by the condition in which each site shows higher abundance, indicating a predominance of serine phosphorylation across comparisons. **(C)** Phosphosite-based pathway enrichment analysis using a pre-ranked *fgsea* against MSigDB Hallmark gene sets, showing normalized enrichment scores (NES) for pathways enriched in EGF or VGF-treated samples. **(D)** Gene Ontology Biological Process (GO: BP) enrichment analysis, using the same pre-ranked approach, highlighting ligand-specific biological processes associated with EGF and VGF-driven phosphorylation patterns.

We performed phosphosite-based pathway enrichment analysis using a pre-ranked approach implemented *via the* fast gene set enrichment analysis *(fgsea)* package [26] against the MSigDB Hallmark gene sets (Fig 2C), as well as Gene Ontology Biological Process analyses (GO: BP) (Fig 2D). These complementary analyses provide a more complete view of the large and complex biological datasets, with Hallmark gene sets representing broad signaling programs and GO: BP identifying more specific biological processes represented among the regulated phosphoproteins.

EGF-associated phosphorylation enriched for Hallmark gene sets related to IL6-JAK-STAT3 and IL2-STAT5 signaling. Representative phosphosites contributing to these patterns included IL6ST S366, which was 14.3-fold higher in EGF than VGF, and AHNAK S5031, which was 4.0-fold higher in EGF. GO analysis also identified EGF-associated processes related to endocytosis and transmembrane transport. Representative phosphosites included DAB2 S401 and CD2AP S510 within the endocytosis gene set, which were 13.6- and 21.0-fold higher in EGF, respectively, and SLC2A1 S226 within transmembrane transport, which was 15.6-fold higher in EGF.

In contrast, VGF-associated phosphorylation showed enrichment of Hallmark MYC targets and peroxisome gene sets, together with GO terms related to carbohydrate catabolism, cytokine regulation, and transcription. Representative VGF-associated sites included PGAM1 S14 within the cellular carbohydrate catabolic process, which was 30.2-fold higher in VGF, and IL1A S87 within cytokine-related processes, which was 69.8-fold higher in VGF. Several phosphosites also contributed to transcription-related GO terms, including MYBBP1A S743, DEK S229, and CAMK2D S330, which were 3.7-, 26.3-, and 15.5-fold higher in VGF, respectively. Representative phosphosites contributing to the major enriched pathways, together with their corresponding phosphorylation changes and effect sizes, are summarized in S1 File. Together, these results show that VGF treatment is associated with enrichment of phosphosite-level signaling toward a set of pathways involved in transcriptional regulation, inflammatory signaling, and metabolic processes that differ from those enriched under EGF stimulation.

### Kinase-substrate and motif analyses identify the ligand-specific signaling biases

To further understand how EGF and VGF induce specific phosphorylation patterns, we performed phosphosite-level signature enrichment analysis using Post Translational Modification Signature Enrichment Analysis (PTM-SEA) against curated PTM signatures database (PTMsigDB) signatures (Fig 3A). In contrast to the gene-level pathway analyses shown in Fig 2C, 2D, this approach evaluates enrichment of predefined phosphosite sets linked to specific kinases or perturbation-derived signaling programs [27]. Each signature represents a defined collection of phosphorylation events associated with a particular kinase activity or experimental perturbation. As shown in Fig 3A, kinase substrate-based signatures (KINASE-PSP), including those linked to AKT and related AGC-family kinases, were preferentially enriched among VGF-associated phosphosites, while EGF-associated phosphosites were enriched for several perturbation-derived (PERT) signatures as well as selected kinase programs, including CDK1 and SGK3. To identify the upstream kinases responsible for these phosphorylation patterns, we performed kinase-substrate enrichment analysis (KSEA) [28,29] (Fig 3B). The kinase activity z-score plot (Fig 3B) shows that EGF treatment results in an enrichment of inferred activity of RSK (RPS6KA1) and PAK1, along with GSK3B and mTOR, which is consistent with canonical EGFR effector engagement. On the other hand, VGF treatment showed higher activity of CDK1 and stress-associated MAPKs, including p38 (MAPK14) and JNK (MAPK8), compared to EGF. RAF1 activity was moderately enriched in EGF-treated samples. Together, these results indicate that although both ligands signal through EGFR, they differentially bias downstream kinase utilization. Because PTM-SEA evaluates enrichment of curated phosphosite signatures [27,30], whereas KSEA infers kinase activity based on substrate-level statistics [28,29,31], these analyses also provide a complementary view on how EGF and VGF shape downstream phosphorylation network.

**Fig 3:**
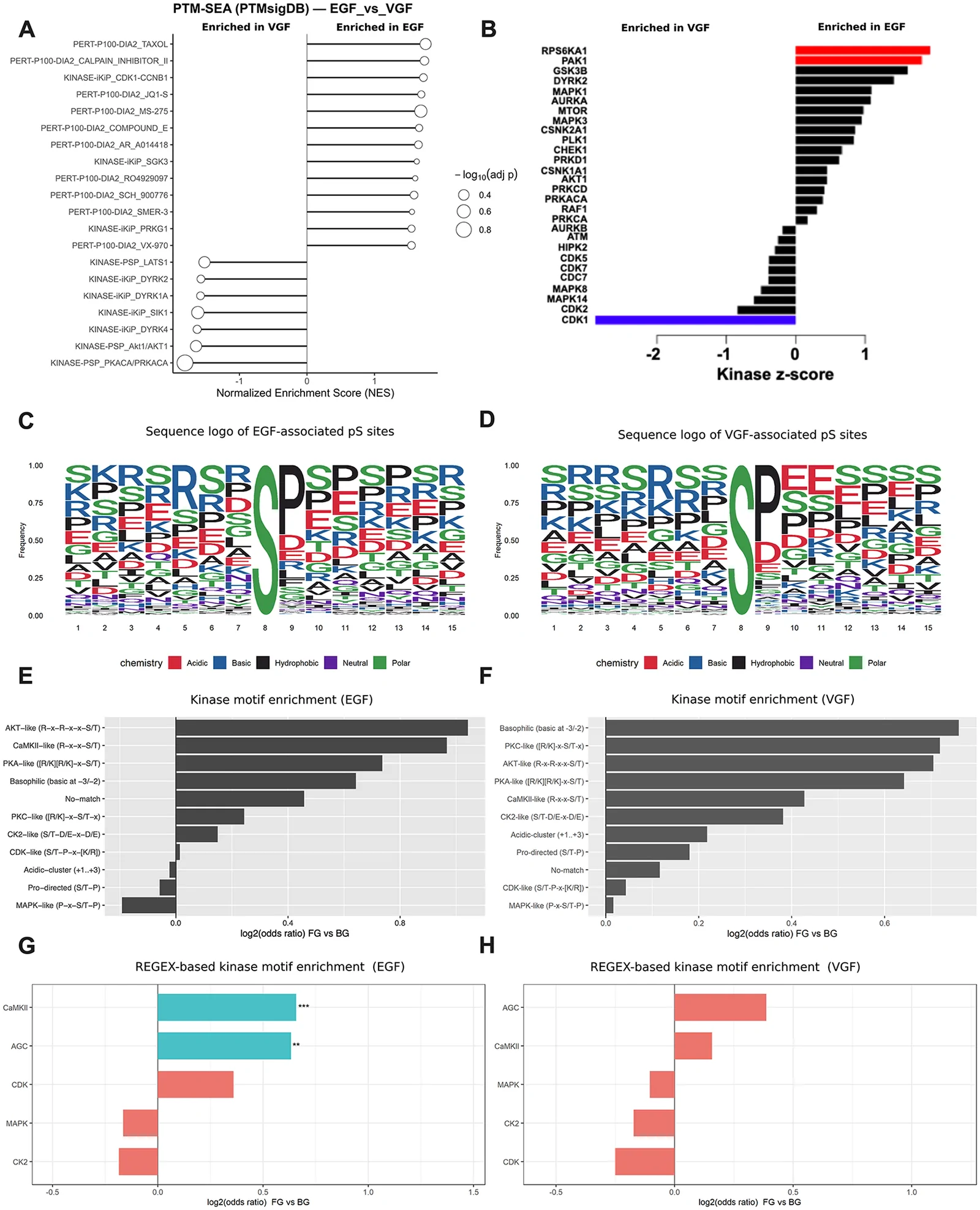
Kinase-substrate and motif analyses reveal ligand-specific signaling biases downstream of EGF and VGF. **(A)** Phosphosite-based signature enrichment analysis using PTM-SEA against PTMsigDB, showing normalized enrichment scores (NES) for phosphosite signatures in EGF versus VGF comparison. Negative NES values indicate enrichment in VGF-treated samples, while positive NES values indicate enrichment in EGF-treated samples. **(B)** Kinase-substrate enrichment analysis (KSEA) showing inferred kinase activity differences between EGF and VGF treatments, plotted as kinase z-scores using the following parameters (p-value cutoff = 0.05, substrate count cutoff = 5). **(C, D)** Sequence logos of serine-centered (pS) phosphorylation sites using 15-mer windows for phosphosites enriched in EGF (**C**) or VGF **(D)**, showing amino acid frequency patterns surrounding the phosphorylation site. **(E, F)** Kinase motif enrichment analysis of serine-centered phosphosites, showing enrichment of known kinase recognition motifs (log_2_ odds ratio, foreground vs background) for EGF-enriched (**E**) and VGF-enriched (**F**) sites. **(G, H)** Motif classification of serine-centered phosphosites into kinase families based on predefined sequence patterns (REGEX), summarizing enrichment across major kinase groups for EGF-enriched (**G**) and VGF-enriched (**H**) sites.

We analyzed serine-centered (pS) phosphorylation sites using 15-mer amino acid sequence windows (Fig 3C-H). Amino acid frequency logos (Fig 3C, 3D) demonstrated enrichment of proline at the + 1 position in both conditions, consistent with engagement of proline-directed kinases. The upstream regions in both conditions showed enrichment for basic residues, indicative of basophilic kinase recognition, although subtle differences in overall residue composition were observed between EGF- and VGF-associated sites. To further classify these patterns, we performed a kinase motif enrichment analysis (Fig 3E, 3F). As shown in Fig 3F, VGF-associated serine sites were enriched primarily for basophilic motifs, including AKT-, PKA-, and PKC-like signatures. EGF-associated serine sites (Fig 3E) exhibited stronger enrichment of AGC- and CaMKII-like motifs, along with modest enrichment of CDK-type proline-directed motifs, while MAPK-like motifs contributed minimally. To further corroborate these findings, phosphorylation site sequences were classified into kinase families using predefined regular expression (REGEX) motif patterns (Fig 3G, 3H). REGEX family-level motif summaries show that both ligands favored basophilic kinase activity at serine residues, but with stronger amplitude under EGF treatment.

We extended this analysis to threonine-centered (pT) phosphorylation sites (S3 Fig). Although pT sites represented a smaller subset of the regulated phosphoproteome, they showed more pronounced ligand-specific differences in motif enrichment than pS sites (S3A Fig, S3B Fig). VGF-associated threonine sites showed strong enrichment of proline at the + 1 position, consistent with proline-directed kinase activity, whereas EGF-associated sites displayed enrichment of both proline-directed as well as basophilic features. Kinase motif enrichment analysis (S3C Fig, S3D Fig) demonstrated that VGF-associated threonine sites were preferentially enriched for proline-directed and MAPK-like motifs, with reduced representation of AKT- and CK2-associated motifs. EGF treatment induced robust enrichment across multiple motif classes, including MAPK-, AKT-, CK2-, CaMK-, and CDK-associated signatures (S3C Fig). REGEX family-level summaries (S3E Fig, S3F Fig) highlighted the magnitude of this effect, with MAPK and AGC families exhibiting particularly strong enrichment under EGF stimulation.

To assess how kinase and motif differences translate into pathway-level outcomes, we examined relative phosphorylation changes at individual phosphosites within core components of the EGFR-MAPK and PI3K-Akt-mTOR pathways (S3G Fig). The heatmap shows ligand-specific differences in both the magnitude and direction of phosphorylation across the tested pathways. EGF treatment showed relatively higher phosphorylation of multiple downstream effector sites, including EIF4EBP1 and MAP2K2 phosphosites. In contrast, VGF treatment showed preferential phosphorylation at select upstream components, including several RAF1 residues. Regulatory nodes such as RICTOR and RPTOR exhibited similar or modestly increased phosphorylation patterns in VGF-treated cells compared with EGF-treated cells. In addition to the stronger activation of the mTORC1-S6K1 axis upon EGF treatment in our previous study [20], our ongoing targeted Western blotting analysis of representative phosphoproteins was also consistent with the ligand-specific differences. We observed modest but relatively greater AKT S473 phosphorylation in EGF treated conditions compared with VGF treatment (S3H Fig). The subtle differences observed at these individual phosphosites likely reflect the convergence of multiple upstream pathways on core MAPK and PI3K-AKT components.

In summary, although both ligands signal through EGFR, they induce distinct downstream phosphorylation architectures (Table 1). VGF treatment was associated with a more selective motif enrichment characterized by basophilic serine phosphorylation and proline-directed threonine signaling and stress-associated MAPKs such as p38 and JNK, compared to EGF treatment. In contrast, EGF drives a broader, multi-branch motif enrichment that is simultaneously associated with enrichment of MAPK-, AGC-, CaMK-, and CK2-linked signatures, consistent with classical growth factor signaling [32,33]. Together, these findings indicate that VGF and EGF differentially modulate EGFR signaling through distinct phosphorylation patterns, kinase motif enrichment, and inferred downstream kinase activity.

**Table 1:** Summary of phosphosite sequence motif enrichment patterns associated with EGF and VGF treatment.

Motif Category	VGF-associated (pS)	VGF-associated (pT)	EGF-associated (pS)	EGF-associated (pT)
Basophilic (AGC/CaMK)	High enrichment (~0.7)	Near-neutral/ slight depletion	High enrichment (~0.8–1.0)	Very high enrichment (~1.8)
AKT-like	Moderate enrichment (~0.7)	Reduced	Very high enrichment (>1.0)	Very high enrichment (>2.0)
Pro-directed (CDK)	Near-neutral	Moderate depletion	Mild enrichment (~0.35)	Moderate enrichment (~0.7)
MAPK-like	Slight depletion	Moderate enrichment	Mild depletion (~−0.2)	Very high enrichment (~2.5)
Acidophilic (CK2)	Moderate enrichment	Reduced	Moderate enrichment	Strong enrichment (~1.4)
Overall motif pattern	Basophilic-biased pattern	Selective, residue-dependent	Broad basophilic enrichment	Strong, Multi-pathway enrichment

Note: Values are approximate log2(odds ratio) of motif enrichments interpreted from Fig 3 (pS) and S3 Fig (pT).

### Transcriptomic responses support phosphoproteomic divergence

Because many of the differentially phosphorylated proteins participate in transcriptional regulation, we used transcriptomics to examine whether these ligand-specific signaling differences are also associated with distinct transcriptional readouts. To this end, we performed RNA sequencing on serum-starved HFFs treated with EGF, VGF, or vehicle for 4 and 8 hours (S4A Fig). PCA analysis shows that the VGF- and EGF-treated groups form separate clusters from the vehicle group and from each other (S4B Fig, S4C Fig), indicating distinct transcriptional responses to VGF and EGF treatments. Consistently, volcano plots show clear and peptide-specific separation in gene expression upon VGF and EGF treatment at both time points (S4D Fig, S4E Fig). Pathway-level transcriptional response analyses indicate that VGF and EGF elicit distinct transcriptional responses (Fig 4A, 4B), as evidenced by the clear separation of the samples. At 4 h, VGF treatment resulted in relatively higher activation of E2F/G1-S and G2/M pathway genes in our RNA-seq analysis (Fig 4A), providing independent functional support for the robust CDK1 and CDK2 kinase activities inferred by KSEA (Fig 3B). Consistently, EGF treatment resulted in relatively higher activation of pathways linked to canonical growth-factor output, including PI3K-Akt-mTOR, mTORC1 signaling, and glycolysis (Fig 4A), providing orthogonal support to the distinct kinase activity profiles predicted by KSEA (Fig 3B). VGF showed lower activity across several canonical anabolic and growth-associated programs compared to EGF at 8h (Fig 4B), particularly within cell-cycle programs including E2F targets and the G2/M checkpoint. EGF showed stronger enrichment of proliferative transcriptional programs. VGF-treated cells remained closer to vehicle treatment across multiple pathways, indicating that the difference is primarily driven by stronger activation in the EGF treatment. Together, these data indicate that EGF sustains broader activation of downstream transcriptional programs over time, whereas VGF elicits a more limited or transient transcriptional response.

**Fig 4:**
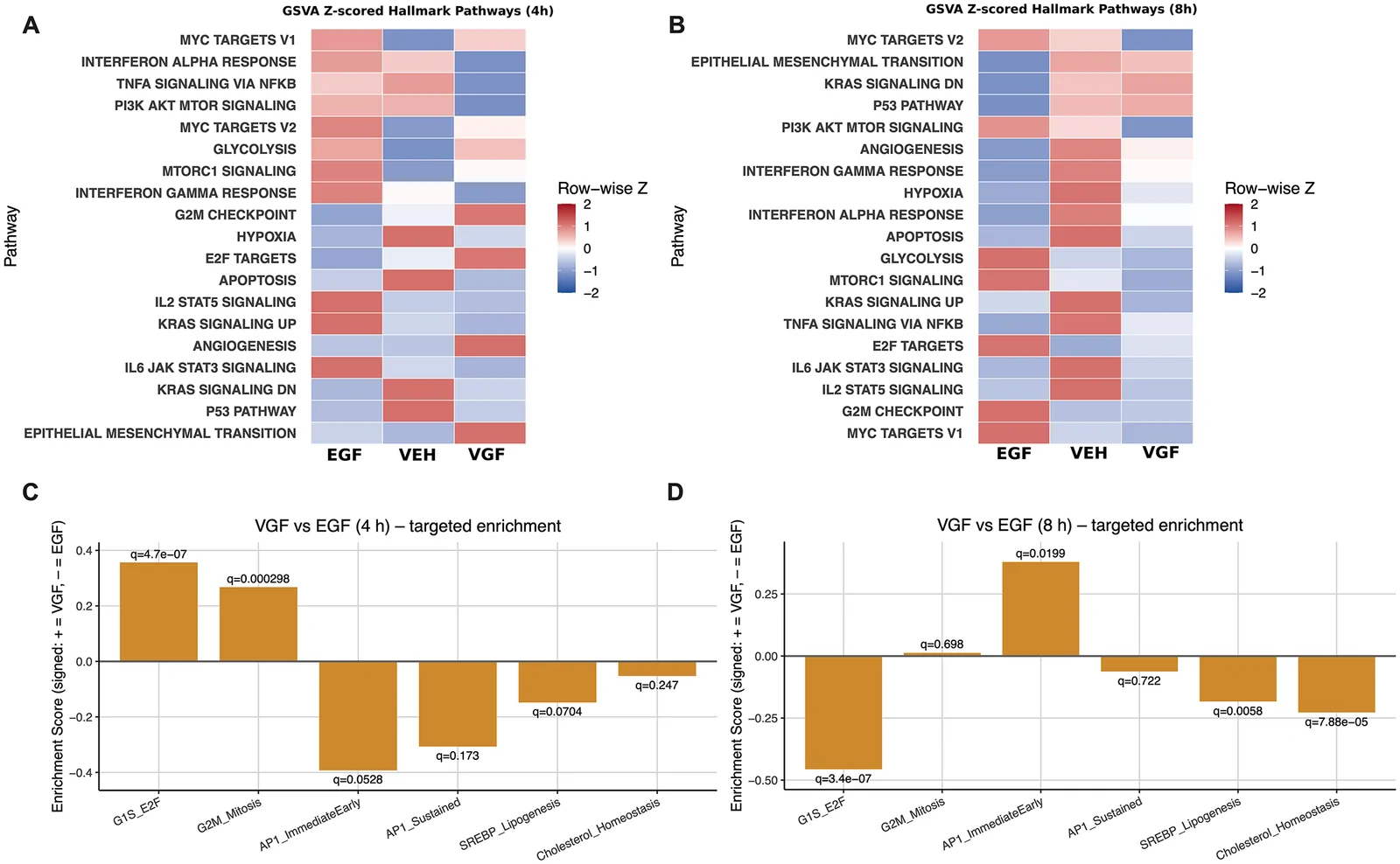
Transcriptomic responses reveal ligand-specific and time-dependent differences between EGF and VGF signaling. **(A, B)** Gene Set Variation Analysis (GSVA) of RNA-seq data showing row-wise Z-scored enrichment of MSigDB Hallmark pathways across vehicle, EGF, and VGF-treated HFFs at 4 h (**A**) and 8 h **(B)**. Pathway activity patterns show clear separation between EGF and VGF treatments, with distinct transcriptional programs observed at each time point. **(C, D)** Targeted enrichment analysis of selected EGFR-associated transcriptional outputs, including cell cycle (G1S_E2F, G2M_Mitosis), MAPK-driven transcription (AP-1 immediate-early and sustained), and PI3K-Akt-mTORC1-linked anabolic programs (SREBP lipogenesis and cholesterol homeostasis), comparing VGF versus EGF at 4 h (**C**) and 8 h **(D)**. Enrichment scores are shown with corresponding q-values indicating statistical significance.

To complement the global GSVA maps with a focused set of EGFR readouts, we performed a targeted enrichment analysis of (i) proliferative cell-cycle modules (G1/S_E2F and G2M_Mitosis), (ii) MAPK-driven transcriptional programs (AP-1 immediate-early and sustained), and (iii) PI3K-Akt-mTORC1-linked anabolic programs (SREBP-dependent lipogenesis and cholesterol homeostasis). These readouts (S2 File) represent well-established downstream transcriptional outputs of EGFR signaling [33], and provide a framework for comparing ligand-specific temporal responses. At 4 h, VGF showed significantly higher enrichment of cell-cycle programs (G1S_E2F and G2M_Mitosis), whereas EGF preferentially activated MAPK-driven transcriptional outputs, including AP-1 immediate-early and sustained programs (Fig 4C). By 8 h, this pattern was shifted. EGF sustained stronger proliferative (G1S_E2F) and anabolic (SREBP-linked lipogenesis and cholesterol homeostasis) programs, while VGF exhibited higher AP-1 immediate-early activity than EGF treatment (Fig 4D). This transcriptional dynamics also closely aligns with the ligand-specific signaling bias observed at the phosphoproteomic analysis (S3G Fig), and our previous studies [20,22]. Together, these findings not only provide orthogonal support for the phosphoproteomics dataset but also indicate that VGF does not simply mirror EGF signaling over time; rather, it drives a time-dependent, distinct transcriptional response.

### Integrated analysis of phosphoproteomics and transcriptomics separates phospho-driven and RNA-driven pathway modules

To integrate the phosphoproteomic and transcriptomic datasets, we used ActivePathways [34,35], which combines statistical evidence from the independent omics dataset to identify biological pathways supported by phosphoproteomic data alone, RNA-seq data alone, or both (Fig 5A). Importantly, this approach does not directly compare RNA abundance with protein phosphorylation or infer causal relationships between them. Instead, it identifies pathways that receive independent statistical support from phosphoproteomic data, transcriptomic data, or both. We used Reactome gene sets for this multi-omics integration because they are well-suited for mapping signaling and regulatory pathways supported by both phosphorylation and gene expression data [34–36].

**Fig 5:**
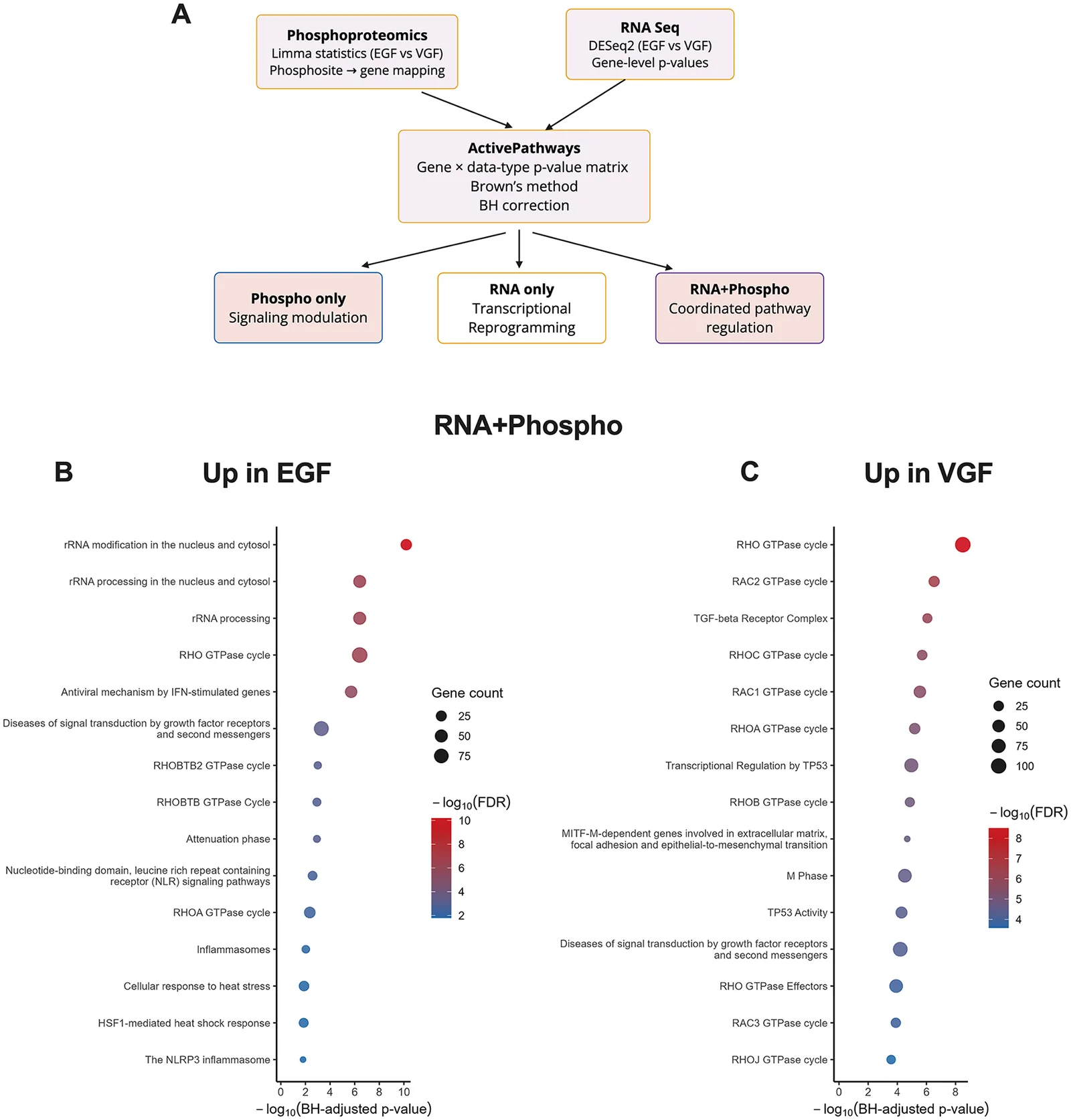
Multi-omics integration separates phosphorylation-driven, transcription-driven, and coordinated pathway programs downstream of EGF and VGF. **(A)** Schematic overview of the multi-omics integration workflow. Phosphoproteomics (limma-derived phosphosite statistics mapped to genes) and RNA-seq (DESeq2-derived gene-level p-values from 4h data) were integrated using ActivePathways with Reactome gene sets, combining evidence across data types using Brown’s method with Benjamini–Hochberg (BH) correction. Pathways were classified as supported by phosphoproteomics only, RNA-seq only, or both (RNA+phospho). **(B, C)** Bubble plots showing significantly enriched Reactome pathways (BH-adjusted p-value) supported by both RNA-seq and phosphoproteomics (RNA+phospho), for pathways upregulated in EGF (**B**) or VGF (**C**) relative to the other condition. Bubble size represents gene set size and color indicates significance (−log_10_ FDR). Fig 5A *Created in BioRender. Pant, A.* (*2026*) https://BioRender.com/282am8o.

In the pathways supported exclusively by phosphoproteomics (S5A Fig), EGF-enriched pathways clustered around pre-mRNA processing/splicing, clathrin-mediated endocytosis, multiple Rho-family GTPase cycles, and ALK-related signaling. This indicates that, compared to VGF, EGF shows greater phosphorylation of proteins annotated to these pathways, with the signal driven at the phosphosite level and not requiring corresponding transcript-level changes. Pathways supported exclusively by phosphoproteomics that were enriched in VGF (S5B Fig), centered on Rho-family signaling (including Rho-PAK modules), growth factor-related signaling, and nuclear regulatory programs involving Notch1, TP53 activity, and epigenetic regulation. These results indicate that, compared with EGF, VGF shows higher phosphorylation of proteins mapped to these pathways, driven primarily by phosphosite-level differences.

Pathways supported exclusively by RNA-seq in EGF (S5C Fig), were dominated by interferon and innate immune signaling, including TLR cascades, cytokine signaling, and antiviral response programs, along with chaperone-mediated protein folding pathways. This indicates that these EGF vs VGF differences are supported by gene expression changes alone and are not detected as phospho-supported pathways in the phosphoproteomic dataset. In the pathways supported exclusively by RNA-seq (S5D Fig), VGF-enriched pathways centered on extracellular matrix organization, DNA replication, cell-cycle progression, and replication stress responses were enriched. This indicates that VGF is associated with distinct transcriptional programs in these pathways, independent of a strong phosphosite-level pathway support.

In the pathways supported by both RNA and phosphoproteomics analysis, EGF-enriched pathways included rRNA processing and ribosome biogenesis, Rho-family GTPase signaling, antiviral/IFN-related programs (including inflammasomes such as NLRP3), and cellular stress responses such as the heat shock pathway. This shows that EGF is preferentially associated with these pathway modules with support from both phosphorylation and gene expression (Fig 5B). VGF-enriched pathways included multiple Rho and Rac GTPase cycles, TGF-β receptor signaling, TP53-linked transcriptional regulation, EMT-related programs, and M-phase cell-cycle processes (Fig 5C). This indicates that VGF is preferentially associated with these modules with coordinated support across both phosphoproteomic and transcriptomic datasets. Overall, these analyses show that EGF and VGF-associated differences map to distinct Reactome pathways that are supported at the different levels of phosphorylation alone, transcription alone, or both (Table 2). EGF-associated pathways span all three levels of regulation and encompass a broad range of processes, including ribosome-related processes, immune signaling, and stress responses. On the other hand, VGF-favored pathways are more focused, with repeated enrichment of Rho/Rac signaling, extracellular matrix remodeling, TP53 and epigenetic regulation, and cell-cycle-related processes. It is important to note that the pathway themes summarized in Table 2 represent Reactome gene sets identified by ActivePathways and indicate pathways that are relatively enriched in each dataset rather than direct activation of individual signaling pathways or receptors.

**Table 2:** Summary of the major pathway themes across evidence classes (phospho-only, RNA-only, RNA+phospho) for EGF- and VGF-associated enrichment.

Evidence class	EGF-associated enriched Reactome pathway themes	VGF-associated enriched Reactome pathway themes
**Supported by Phosphoproteomics**	RHO-family GTPase signaling; clathrin-mediated endocytosis; ALK signaling; mRNA splicing	RHO/PAK signaling; VEGF/EGFR signaling; TP53 acetylation; Notch signaling; epigenetic regulation
**Supported by RNA-seq**	Interferon and TLR signaling; cytokine signaling (IL-4/IL-13); rRNA processing; innate immune pathways	Extracellular matrix organization; collagen biosynthesis; DNA replication; cell-cycle progression; TGF-beta signaling
**Supported by both datasets**	rRNA processing; RHO GTPase signaling; interferon responses; inflammasome pathways; stress responses (HSF1)	RHO/RAC GTPase signaling; TGF-beta receptor signaling; TP53 transcriptional regulation; EMT-related programs; M-phase

### VGF and EGF treatments differentially influence VACV response to different inhibitors and plaque formation

To determine whether the distinct signaling programs identified in our omics analyses were also associated with functional consequences during infection, we performed virus replication and plaque formation assays. In vehicle treated conditions, VGF-deleted VACV (vΔVGF) showed approximately 25% reduction in viral titer compared with wild-type (WT)-VACV, although the difference did not reach statistical significance. Pharmacological inhibition of EGFR with afatinib [37] reduced replication of both WT-VACV and vΔVGF, by 94% and 98% respectively, with a 79% inhibition for vΔVGF virus compared to WT-VACV (Fig 6A). These findings suggest that VGF contributes to EGFR-dependent signaling during WT-VACV infection, whereas the absence of VGF renders virus replication more susceptible to pharmacological inhibition of EGFR.

**Fig 6:**
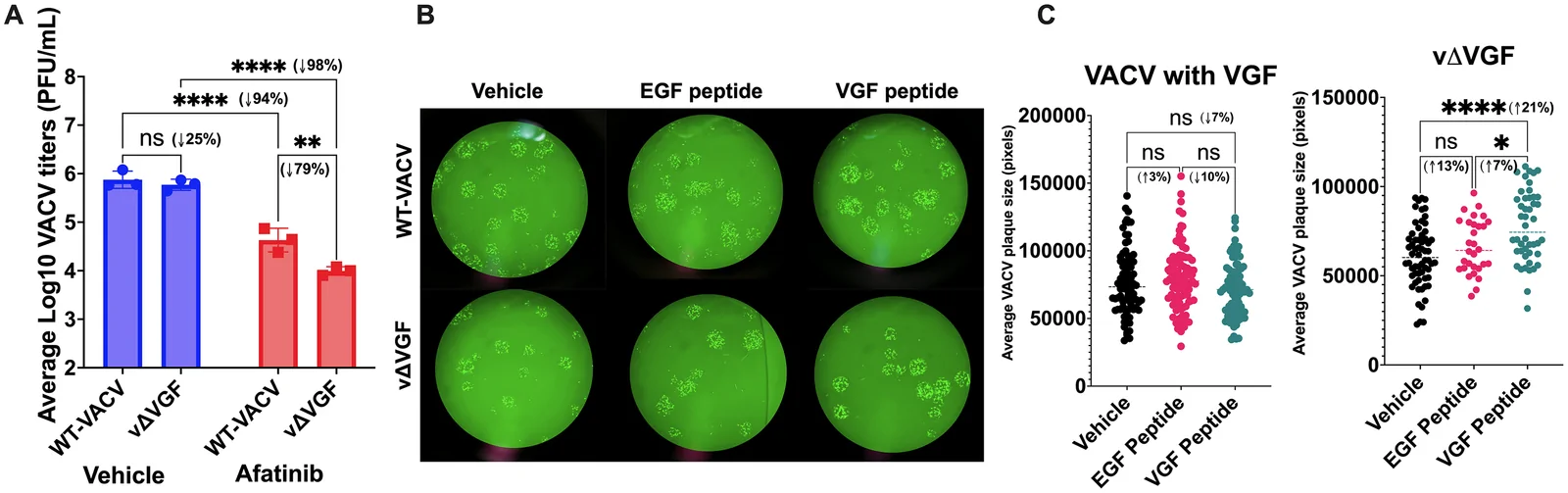
VGF and EGF-dependent effects on VACV replication and plaque formation. **(A)** Viral replication assay in HFFs. Cells were serum starved for 48 h and infected with WT-VACV or vΔVGF at MOI 0.01 in the presence or absence of the EGFR inhibitor (afatinib 3 µM). Virus titers were measured at 48 h post-infection (hpi) using a plaque assay. **(B)** GFP-plaque size measurement in BS-C-1 cells. Cells were serum-starved for 48 h and infected with 50 PFU/well of recombinant GFP-expressing VACVs, with or without the VGF-encoding (C11R) gene. At 1 h post-infection (hpi), starvation media containing 0.5% methylcellulose with vehicle, 5 µg/mL EGF, or 5 µg/mL VGF peptide was added. Representative images were captured at 24 hpi. Images are representative of at least five fields from three independent biological replicates. **(C)** Quantification of plaque size from **(B)**, shown as average plaque diameter (GFP pixels) for VACV with VGF or vΔVGF under each treatment condition. Percentages indicate the effect sizes calculated from the mean PFU/mL values in 6A and mean plaque size (pixels) in 6C. Downward (↓) and upward (↑) arrows indicate percent decrease and increase, respectively, between the indicated groups. The error bars in Fig 6A indicate the standard deviation of the at least three independent biological replicates. Statistical significance is indicated as follows: ns, not significant; *P < 0.05; **P < 0.01; ****P < 0.0001.

Previous studies have established a major role of VGF-induced EGFR signaling in VACV spread [16]. To examine the effect of EGF and VGF treatment on viral spread, we examined plaque formation in VACV with or without VGF (vΔVGF) upon treatment with vehicle, EGF, or VGF peptide using green fluorescent protein (GFP) expression as a readout. The GFP is expressed by both viruses under the same p11 viral promoter. In the cells infected with GFP-expressing VACV with VGF, the plaque morphology was mostly similar across all treatment conditions at 24hpi (Fig 6B). In the cells infected with GFP-expressing WT-VACV, mean plaque size was modestly smaller following EGF (3%) or VGF (10%) treatment compared with vehicle, which did not reach statistical significance (Fig 6C). In contrast, the vΔVGF VACV infection showed treatment-dependent differences. VGF peptide treatment increased mean plaque size by approximately 21% compared with vehicle and by 7% compared with EGF, whereas EGF increased mean plaque size by approximately 13% compared with vehicle and this difference was not statistically significant (Fig 6C). In addition, plaques formed in the presence of VGF peptide treatment were more circular and well-defined, while plaques formed with EGF or vehicle appeared less organized and with more diffused and irregular edges (Fig 6B). Together, these findings suggest that VGF peptide treatment enhances plaque formation in the vΔVGF background and promotes more organized viral spread.

## Discussion

In this study, we show that although vaccinia viral and cellular growth factors engage the shared EGFR receptor, they transmit downstream signaling in different ways. Our integrated phosphoproteomic and transcriptomic analyses indicate that the differences emerge at downstream branch points in kinase utilization and signal transduction. EGF stimulation is associated with broader engagement of MAPK, AGC, CaMK, and CK2-linked kinase programs and sustained activation of anabolic and proliferative transcriptional outputs, consistent with classical growth factor signaling. Interestingly, VGF biases EGFR towards a more selective pattern of phosphorylation, which is accompanied by more selective and temporally regulated transcriptional programs enriched for Rho/Rac signaling, TP53 regulation, and extracellular matrix-associated cell remodeling. Multi-omics integration further demonstrated that these differences occur at multiple levels, with certain pathway modules supported predominantly by phosphorylation, others by transcription, and a subset by coordinated regulation at both levels. Together, these findings support a working model (Fig 7) in which VGF does not simply mimic EGF signaling but reconfigures the downstream EGFR signaling toward a qualitatively distinct and time-dependent regulatory state, potentially enabling viral manipulation of host signaling networks without simply recapitulating canonical growth factor programs.

**Fig 7:**
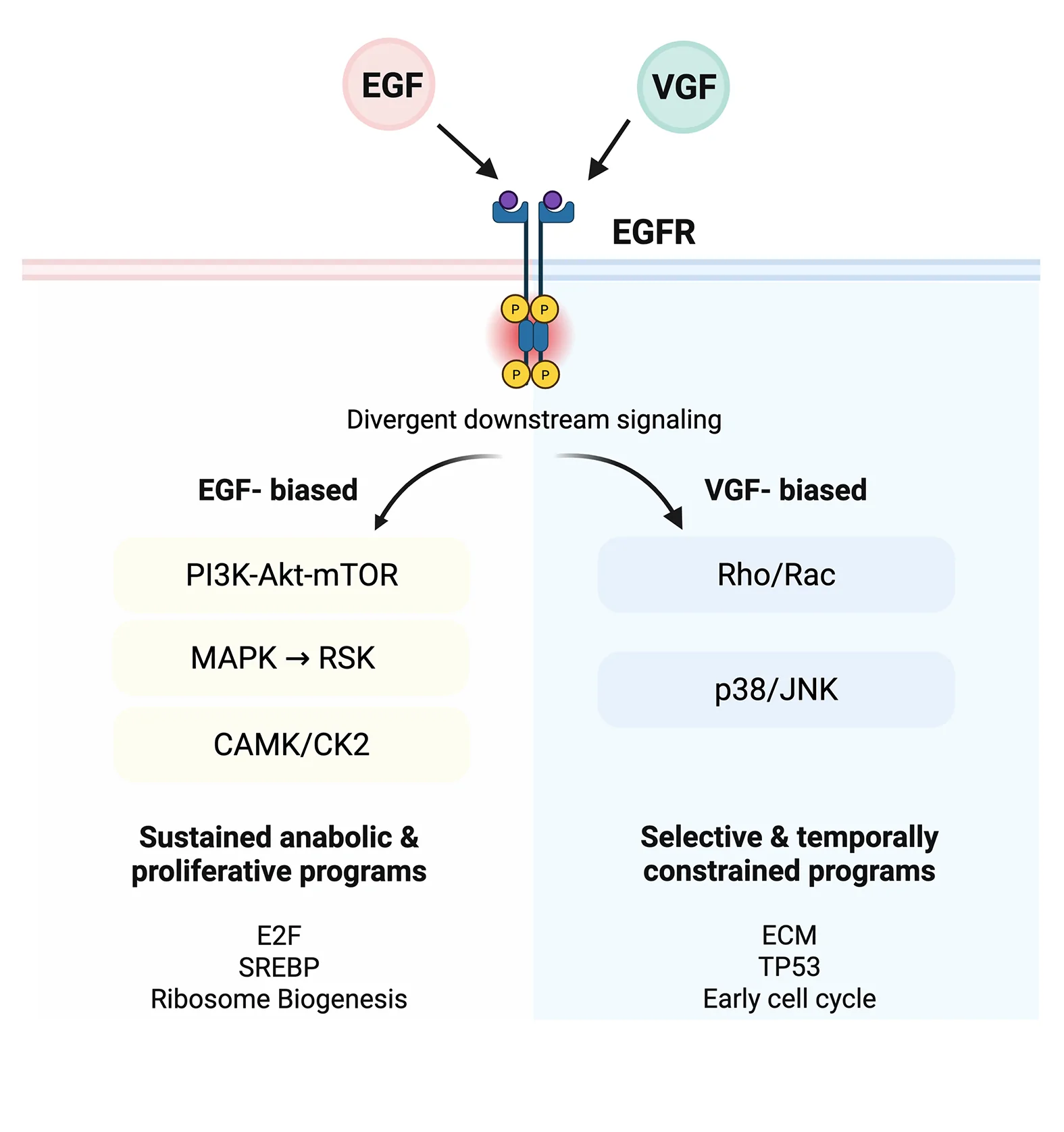
A working model of ligand-biased EGFR signaling driven by EGF and VGF. Schematic summarizing the key findings of this study. Although both EGF and VGF activate EGFR, they induce distinct downstream signaling outputs. EGF preferentially engages PI3K-Akt-mTOR and MAPK-RSK pathways, which lead to sustained anabolic and proliferative programs, including E2F, SREBP, and ribosome biogenesis. In contrast, VGF promotes a more selective signaling profile characterized by Rho/Rac and stress-associated MAPK pathways (p38/JNK), which results in temporally constrained transcriptional programs linked to extracellular matrix remodeling, TP53 signaling, and early cell-cycle responses. *Created in BioRender. Pant, A. (2026)* https://BioRender.com/zuk5qgl.

Our previous study showed that VGF-peptide induces robust EGFR phosphorylation, exceeding that induced by EGF treatment [20]. However, the downstream propagation of canonical EGFR signaling pathways observed in this study differs substantially between the ligands. These findings suggest that the magnitude of receptor phosphorylation does not necessarily predict the pattern or strength of downstream signaling. Previous work on different poxvirus growth factors has shown that strong biological responses can occur despite lower receptor-binding affinity [38], in part by altering receptor downregulation and trafficking. Reduced receptor degradation can prolong receptor phosphorylation and signaling without necessarily producing the same downstream signaling effects as that induced by cellular ligands. In this context, robust EGFR phosphorylation observed under VGF stimulation in our previous studies [20,22] may reflect altered receptor handling rather than equivalent downstream signaling. This idea is consistent with broader studies of EGFR ligands, which show that different ligands can elicit distinct cellular responses, including differences in proliferation, differentiation, and motility [39–43]. Individual ligands have also been shown to generate distinct downstream signaling profiles and *in vivo* phenotypes [41,44,45]. Our model is also consistent with emerging studies on receptor tyrosine kinases [46], in which different ligands can stabilize distinct receptor conformations or trafficking states, thereby influencing adaptor recruitment and downstream outputs. While we did not directly examine receptor assembly or trafficking, the differential signaling patterns observed in our study suggest that VGF and EGF may engage EGFR signaling complexes in distinct spatial or temporal contexts [44,46,47]. Kinase motif analysis further supported these differences. Although serine sites were much more abundant (Fig 2B), the differences between EGF and VGF were more pronounced at threonine sites, with EGF showing stronger enrichment of MAPK-, AKT-, and CK2-associated motifs, while VGF showed a more limited and distinct pattern (Fig 3 and Table 1). In this regard, VGF may function as a context-dependent modulator of EGFR signaling during infection, potentially representing an evolutionary strategy by which poxviruses reshape host signaling without merely amplifying canonical growth pathways.

Our RNA-seq data further show that VGF signaling differs from EGF in how downstream responses are sustained over time. EGF maintained proliferative and anabolic transcriptional programs, whereas VGF induced more transient and selective responses. Early cell-cycle-related transcriptional programs induced by VGF declined at later time points, suggesting that the initial signal is not sustained at the transcriptional level. This pattern is consistent with the more limited engagement of key downstream branches, such as mTOR and MAPK, which are required to sustain proliferative transcription [48,49]. At the same time, the relative increase in inferred AP-1 activities at later time points also raises the possibility that some VGF-driven outputs are temporally shifted or delayed rather than being simply reduced. Together, these findings suggest that VGF induces a distinct signaling program rather than fully recapitulating the rapid and sustained EGFR signaling induced by EGF.

Our findings are consistent with the previous studies showing an important role of VGF in promoting VACV spread and cell motility [12,16,17]. Deletion of VGF resulted in a modest reduction in viral titer under vehicle-treated conditions, whereas EGFR inhibition strongly reduced replication of both WT and vΔVGF VACV (Fig 6A), supporting an important role for EGFR signaling during infection. The effect of VGF was more apparent in plaque formation, which is in alignment with its role in enhancing plaque formation both *in vivo* and *in vitro* [16,17,21]. These findings also suggest that plaque size may be a better indicator of VGF’s role in VACV replication rather than viral titers alone. In the vΔVGF background, exogenous VGF significantly increased plaque size compared with vehicle and EGF treatment (Fig 6B-C), whereas EGF did not significantly increase plaque size compared with vehicle. VGF-treated plaques also appeared more circular and well-defined, although these morphological differences were not quantified. The distinct signaling responses identified in our phosphoproteomic and transcriptomic analyses may, therefore, contribute to cellular processes that support efficient viral spread rather than simply increasing overall virus production. These findings also extend our previous studies showing that VGF promotes metabolic programs involving the TCA cycle, neutral lipids, and pyrimidine synthesis during VACV infection [20–22]. Together with the cytoskeletal and membrane-trafficking processes identified in this study (Table 2), these observations suggest that VGF may coordinate both metabolic and signaling responses that support the biosynthetic and cellular requirements for efficient virus replication and spread.

From the perspective of the vaccinia virus, this type of selective signaling may provide several advantages. By engaging EGFR while biasing downstream pathway utilization, VGF may create an environment that supports viral replication without full commitment towards canonical growth factor-driven anabolic processes. Such selective rewiring could enable the virus to exploit host signaling machinery while minimizing cellular states that are energetically costly or immunologically unfavorable. Because VGF is secreted, these signaling effects extend beyond infected cells and influence neighboring cells within the local tissue environment. In this way, VGF can help create an environment in the cell that is more permissive for viral dissemination before infection occurs. More broadly, our findings support the idea that viral proteins don’t just amplify existing host signaling but also selectively modulate the host pathways to create a functionally distinct environment that favors viral replication [19,50,51].

These findings of our phosphoproteomic studies are also consistent with previous studies showing that poxvirus infection induces broad alteration of host signaling networks [52–54]. Studies using F10- or H1-deficient VACV identified phosphorylation changes linked to multiple cellular processes, including cytoskeletal remodeling, membrane trafficking, and cellular metabolism [52,53]. These studies primarily focused on phosphorylation associated with specific viral enzymes rather than global differences between uninfected and WT VACV-infected cells. However, these viruses retained VGF, which implies some of the host signaling changes observed in those datasets may also reflect VGF-dependent signaling. More recently, phosphoproteomic profiling of MPXV-infected primary HFFs identified changes in MAPK and mTOR signaling, PAK proteins, cytoskeletal organization, metabolism, and cell morphology and motility [54], several of which overlap with the major signaling themes identified in our study. These similarities support the biological relevance of our findings. However, direct comparison is limited by differences in viruses, experimental conditions, and the contribution of many other viral proteins during infection. Future studies using WT and vΔVGF VACV in primary HFFs will therefore be important to define the specific contribution of VGF in remodeling host signaling networks during VACV infection.

By using peptide stimulation in uninfected cells, our data provide a controlled framework for comparing ligand-specific signaling by reducing confounding effects of viral kinases, host stress, and infection-associated transcriptional responses. However, EGFR signaling varies across cell types and differentiation states [55]. Therefore, the magnitude or direction of signaling bias may differ in other contexts. In the future, extending these studies to infected cells will also be important to better understand how VGF-driven signaling interacts with viral gene expression and virus-induced signaling during infection. Although our study demonstrates that VGF and EGF induce distinct downstream signaling responses, the molecular basis for this signaling divergence remains to be determined. One possibility is that VGF and EGF differentially regulate EGFR trafficking, including receptor internalization, recycling, or endosomal signaling, thereby altering the duration and spatial organization of downstream signaling. Future studies on EGFR trafficking will be important for addressing this question. Although the recombinant VGF used in this study was expressed in mammalian cells, differences in recombinant protein preparation, post-translational modifications, or specific activity between EGF and VGF remain a limitation for the direct comparison of the ligands. However, the VGF peptide has been shown to induce EGFR phosphorylation and modulate downstream targets such as CAD and S6K1. These previous findings also showed that strong VGF-induced EGFR phosphorylation does not automatically lead to stronger phosphorylation of downstream targets, which is consistent with the distinct downstream signaling patterns observed in the current study. Further validation of representative signaling pathways, together with dose-dependent comparison of EGF and VGF, will be important to determine the extent to which ligand concentration or specific activity contributes to the observed differences. While individual changes in total protein abundance could not be factored out due to the absence of a parallel global proteomic dataset, our integrated phosphoproteomic and transcriptomic analyses provide a framework to examine ligand-specific EGFR signaling and establish a foundation for future mechanistic studies of VGF function during poxvirus infection.

In summary, our study shows that VGF and EGF, although acting through the same receptor, lead to different downstream outcomes. These findings highlight an important layer of regulation in which ligand identity shapes how signaling is coordinated and how cells respond. More broadly, our work shows that integrated multi-omics approaches can provide a framework for understanding how different ligands acting on the same receptor result in distinct biological outcomes, which opens the door to exploring how these differences shape cell behavior in the context of health and disease.

## Materials and methods

### Cell culture and growth factor treatments

Primary human foreskin fibroblasts (HFFs) were cultured in T175 flasks and subjected to serum starvation to minimize basal growth factor signaling. Cells were incubated for 48 h in starvation medium consisting of DMEM supplemented with 0.1% dialyzed fetal bovine serum, 2 mM L-glutamine, 1 g/L glucose, and penicillin/streptomycin.

Following the starvation, cells were treated for 4 h with either vehicle control (PBS), 5 µg/mL epidermal growth factor (EGF), or 5 µg/mL vaccinia virus growth factor (VGF) peptide, all prepared in starvation medium. The EGF peptide was purchased from Cell Signaling Technologies. The cleaved, secreted form of VGF peptide containing a C-terminal 6 × His tag was expressed from a plasmid encoding the *de novo* synthesized gene and purified using a mammalian expression system in the HD CHO-S cell line at GenScript, as described and characterized previously [20]. The VGF peptide sequence with 6xHis tag used is as follows:

DSGNAIETTSPEITNATTDIPAIRLCGPEGDGYCLHGDCIHARDIDGMYCRCSHGYTGIRCQHVVLVDYQRSENPNTHHHHHH.

At the end of the treatment period, cells were washed twice with PBS, trypsinized, and collected by centrifugation. Cell pellets were flash-frozen in a dry ice-ethanol bath and stored at -80 °C overnight before shipment to Creative Proteomics (Shirley, NY) for downstream phosphoproteomics analysis.

For RNA sequencing sample preparation, HFFs were starved and treated with 5 µg/mL VGF or EGF peptides, or with PBS control, as described above. Total RNA was extracted from the lysates using the Ambion Pure Link RNA Mini Kit according to the manufacturer’s instructions at the indicated time points. Extracted RNA was purified and sent for sequencing to the Molecular Genomics Core at the Institute for Genome Sciences and Society at Texas A&M University.

For phosphoproteomics analysis, four independent biological replicates were generated for each treatment condition (vehicle, EGF, and VGF). Each biological replicate consisted of an independently cultured, treated, and harvested HFF sample that was submitted individually for phosphoproteomic analysis. For RNA-seq, three independent biological replicates were prepared and processed in the same manner.

### Phosphoproteomics sample preparation and mass spectrometry

Phosphopeptide enrichment, LC-MS/MS acquisition, and data processing were performed by Creative Proteomics using proprietary protocols for quantitative phosphoproteomics analysis in the samples (four biological replicates per treatment) prepared as described above. Proteins were extracted, quantified, and digested with trypsin, followed by enrichment of phosphorylated peptides using iron-based immobilized metal affinity chromatography (Fe-IMAC). Enriched phosphopeptides were analyzed by nano-liquid chromatography coupled to tandem mass spectrometry (nano-LC-MS/MS) using a high-resolution Orbitrap platform.

Peptide separation was achieved on a reverse-phase C18 nanocolumn with a linear acetonitrile gradient, and data were acquired in data-dependent acquisition mode. Raw mass spectrometry files were searched against the *Homo sapiens* protein database using MaxQuant software (version 1.6.2.6), with phosphorylation on serine, threonine, and tyrosine residues included as variable modifications. Peptide and protein identifications were filtered using standard false-discovery-rate thresholds. Quantitative phosphoproteomics data were subsequently used for downstream inference of kinase activity, motif enrichment, and pathway analyses.

### Phosphoproteomics data processing and bioinformatic analysis

Phosphorylation site information was extracted from the Phospho (STY) Sites table generated by MaxQuant, and label-free quantification (LFQ) intensities were used for quantitative comparisons. Only confidently localized phosphosites were retained by applying a site localization probability threshold of ≥0.75. To improve data quality, common contaminants, reverse database hits, and phosphosites identified solely by site were removed. Phosphosites were required to have at least two non-missing values per experimental condition to ensure robustness. LFQ intensities were log2-transformed, and normalization was performed under the assumption that most phosphosites remain unchanged across conditions. Data distributions were assessed using MA plots and density plots to confirm appropriate normalization.

Missing values were not imputed. Instead, phosphosites were filtered based on data completeness, and analyses were performed using observed values to avoid introducing artificial signals and to ensure that results more accurately reflect the measured phosphorylation changes.

Differential phosphorylation analysis was performed on filtered phosphosite-level data using the limma package in R (version 4.2.3). Log2 fold changes and associated p-values were calculated for all retained phosphosites, followed by Benjamini-Hochberg correction for multiple testing. Phosphosites with a false discovery rate (FDR) < 0.05 were considered significantly regulated. An additional fold-change cutoff |log2FC| ≥ 1 was used to identify strongly regulated sites for downstream interpretation and visualizations.

Data quality and structure were evaluated using unsupervised analyses, including principal component analysis (PCA), and volcano plots, and hierarchical clustering heatmaps using the ggplot2, pheatmap, and other related packages in R (version 4.2.3). Phosphosite intensities were standardized by calculating row-wise Z-scores across samples. Hierarchical clustering was applied to phosphosites only, while sample columns were retained in their predefined experimental order to facilitate comparison among vehicle-, EGF-, and VGF-treated groups.

For performing the S/T/Y distribution analyses, the ggplot2 package in R was used. Phosphosites were filtered for significance (FDR < 0.05), separated by direction of change, and grouped by the condition with increased phosphorylation. Counts of serine (S), threonine (T), and tyrosine (Y) residues were calculated and visualized as grouped bar plots.

### Kinase activity and pathway enrichment analyses

Functional interpretation of regulated phosphosites was performed using kinase substrate enrichment analysis (KSEA) with KSEAapp [28] based on kinase-substrate relationships curated from PhosphoSitePlus. Custom R scripts were used to generate the dataset in the correct format for input into the KSEApp for analysis. Kinase activity scores were calculated as z-scores based on the distribution of substrate phosphorylation changes. For plotting the graph, a p-value cutoff of 0.05 and a substrate count cutoff of 5 were used.

Post-translational modification set enrichment analysis (PTM-SEA) was performed using PTMsigDB (v2.0.0) [27] and a preranked gene set enrichment framework implemented in the fgsea package in R (version 4.2.3). Phosphosites were ranked based on limma moderated t-statistics, and enrichment was calculated using the fgsea Multilevel algorithm without prior fold-change or significance filtering.

For pathway-level analysis, site-level phosphorylation signals were collapsed to gene-level ranks by selecting the phosphosite with the largest absolute moderated t-statistic per gene. Pre-ranked gene set enrichment analysis was then performed using the fgsea package with gene sets obtained from MSigDB via the msigdbr package, including the Hallmark collection and Gene Ontology Biological Process (GO: BP) terms. Enrichment significance was determined using Benjamini–Hochberg correction, with adjusted p-values < 0.05 considered significant.

### Motif and sequence analysis

To examine sequence-level features of regulated phosphorylation sites, serine-centered (pS) and threonine-centered (pT) sequences were extracted using 15-mer windows (±7 amino acids) surrounding the modified residue based on the UniProt reference proteome (*Homo sapiens*, UP000005640, Release 2025_01). Amino acid frequency logos were generated using ggseqlogo package in R.

Kinase motif enrichment analysis was performed using curated consensus phosphorylation motifs, and enrichment was calculated as log2(odds ratio) comparing foreground (regulated sites; FDR < 0.05) to background sets consisting of all quantified phosphosites of the same central residue (S or T as indicated). Statistical significance was assessed using Fisher’s exact test with Benjamini–Hochberg correction (adjusted p < 0.05).

In parallel, phosphorylation sites were also classified into kinase families using predefined regular expression (REGEX)-based motif patterns corresponding to major kinase classes (e.g., AGC, CaMK, CDK, MAPK, CK2). This analysis was performed using custom R scripts, enabling family-level comparison of motif preferences among different ligand treatment conditions.

### RNA sequencing sample preparation and data analysis

The samples for RNA sequencing were prepared using the Illumina Stranded mRNA Prep, Ligation kit. RNA concentration and library quantification were determined using a Qubit fluorometer. RNA integrity prior to library preparation and final library quality and size distribution were assessed using the Agilent TapeStation 4200. Sequencing was performed on the Illumina NextSeq 2000 platform.

Raw RNA-seq reads were aligned to the reference genome (GRCh38.p14, GENCODE Release 47) using HISAT2. Once aligned, the reads were quantified at the gene level by using the featureCounts function from the Subread package using the GENCODE Release 47 GTF annotation file. The resulting count matrix was imported into R, where genes with low total counts (< 10 across all samples) were removed prior to analysis. Differential expression analysis was conducted using the DESeq2 package, and variance-stabilizing transformation (VST) was performed using the vst function with blind = TRUE for downstream analyses. Genes were considered significantly differentially expressed if they had an adjusted p-value (padj) < 0.05 and an absolute log_2_ fold change > 1.

Gene set variation analysis (GSVA) was performed on VST-normalized expression data from the indicated time points using the GSVA package in R with Hallmark gene sets obtained from MSigDB via the msigdbr package. For visualization, GSVA scores were summarized across treatment conditions and scaled to generate row-wise z-scores for the heatmap. In addition, targeted gene set analyses were performed using manually curated gene lists representing the biological processes of interest as indicated. Gene sets were compiled based on literature and established pathway annotations, including gene signatures from the Molecular Signatures Database (MSigDB) (S2 File). Differential expression analysis was performed using DESeq2 to obtain gene-level log_2_ fold changes for the VGF versus EGF comparison. For each gene set, an enrichment score was calculated as the mean log_2_ fold change of all genes within the set that were detected in the dataset. Statistical significance was assessed using a one-sample t-test against zero across genes within each set, and p-values were adjusted for multiple testing using the Benjamini–Hochberg method. Adjusted p-values are reported as q-values.

### ActivePathways multi-omics integration of RNA-seq and phosphoproteomics

To identify signaling pathways differentially enriched downstream of EGF and VGF, we applied ActivePathways analysis to perform a directional multi-omics integration of the 4h phosphoproteomics and RNA-seq data [34]. Briefly, the differential expression results from RNA-seq (DESeq2) and gene-level statistics derived from phosphoproteomics were used as inputs. For each modality, gene-level p-values were assigned directionality based on the EGF versus VGF contrast (positive log2 fold changes classified as EGF-up and negative values as VGF-up). When multiple measurements mapped to the same gene, the minimum p-value per gene was retained.

Gene identifiers were harmonized by mapping gene symbols to Entrez Gene IDs using the org.Hs.e.g.,db annotation package. For each direction (EGF-up and VGF-up), modality-specific p-values were compiled into vectors indexed by Entrez ID. Human Reactome pathway gene sets were obtained from the reactome.db package, and pathways containing between 10 and 500 genes were retained for analysis.

Directional pathway enrichment analyses were performed separately for EGF-up and VGF-up gene sets. RNA-seq and phosphoproteomics p-values were combined into a two-column matrix, with missing values assigned a p-value of 1. Integration was performed using the ActivePathways R package with Brown’s method to combine evidence across data types, and multiple testing correction was applied using the Benjamini–Hochberg procedure. Pathways with adjusted p-values < 0.05 were considered significant. Enriched pathways are visualized as bubble plots, with bubble size reflecting the number of overlapping genes and color and shape indicating the contributing data modality.

### Virus infection and plaque size measurement

Human foreskin fibroblasts (HFFs) were serum-starved for 48 h prior to infection in the starvation media. The starvation medium was prepared using 1X DMEM lacking glucose, L-glutamine, L-asparagine, sodium pyruvate, and phenol red, and was supplemented with 0.1% dialyzed FBS (Gibco), 2 mM glutamine, 1 g/L glucose, 100 U/mL penicillin, and 100 µg/mL streptomycin (VWR). For viral replication assays, HFFs were infected with VACV Western Reserve (WR) strain (ATCC VR-1354) or VGF-deleted (vΔVGF) VACV at a MOI of 0.01 in the presence or absence of the EGFR inhibitor afatinib (3 µM). Viral titers were determined at 48 h post-infection (hpi) by plaque assay.

The recombinant VACVs expressing GFP under the viral late p11 promoter, including wild-type VACV and the VGF-deleted virus (vΔVGF), were used in this study and have been described previously [21,56]. To measure plaque size using GFP, BSC-1 cells were serum-starved for 48 h in starvation medium and infected with 50 PFU/well of the indicated VACV strains encoding GFP under the viral late p11 promoter. At 1 hpi, infection media were replaced with starvation media containing 0.5% methylcellulose supplemented with vehicle, EGF (5 µg/mL), or VGF peptide (5 µg/mL). Plaques were imaged at 24 hpi, and plaque size was quantified from GFP fluorescence in Fiji (ImageJ) by measuring the plaque diameter (GFP pixels). Analyses were performed across at least five different fields from at least three independent biological replicates.

### Western blotting analysis

Western blotting was performed as previously described [57], with minor modifications. Briefly, after indicated treatments the cells were lysed using NP-40 lysis buffer, and samples were prepared with sodium dodecyl sulfate (SDS) loading buffer containing 100 mM dithiothreitol (DTT). Samples were heated at 99°C for 5 min and separated by SDS-PAGE. Proteins were transferred to polyvinylidene difluoride (PVDF) membranes using the Trans-Blot Turbo Transfer System (Bio-Rad) for 10 min. Membranes were then blocked with 5% milk in 1 × TBST for 1 h at room temperature and incubated with the indicated primary antibodies in blocking buffer either overnight at 4°C or for 1 h at room temperature.

Following primary antibody incubation, membranes were washed three times with 1 × TBST for 10 min each and incubated with horseradish peroxidase (HRP)-conjugated secondary antibody (Cell Signaling Technology) for 1 h at room temperature. Protein bands were detected using SuperSignal West Femto Maximum Sensitivity Substrate (Thermo Fisher Scientific) and imaged using an Azure 300 chemiluminescence imaging system. Whenever required, membranes were stripped using Restore PLUS Western Blot Stripping Buffer (Thermo Fisher Scientific) and reprobed with additional primary antibodies. The antibodies against phospho EGFR (Y1068), total EGFR, phospho AKT (S473), and total AKT were purchased from Cell Signaling Technology.

### Statistical analysis

Unless otherwise stated, the results are expressed as mean of a minimum of three independent biological replicates, with error bars representing the standard deviation (SD). For the global phosphoproteomics, four biological repeats were used for each condition, and three biological replicates were used for each condition for the transcriptomics. Data processing and statistical evaluations were conducted using GraphPad Prism (v10.2.3) and Microsoft Excel (v16.43). To determine statistical significance between two groups, a two-tailed paired t-test was applied. For comparisons involving more than two groups, a one-way ANOVA was utilized, followed by Tukey’s HSD or Dunnett’s post-hoc tests as appropriate. Interactions between infection status (infected vs. uninfected) and treatment type (vehicle vs. compound) were analyzed via two-way ANOVA with a Tukey’s HSD post-test.

Significance levels are indicated as follows: ns: P > 0.05; *: P ≤ 0.05; **: P ≤ 0.01; ***: P ≤ 0.001; ****: P ≤ 0.0001

## Supporting information

S1 Fig Quality control and normalization of phosphoproteomics data. (**A**) Pearson correlation heatmap of phosphoproteomic samples showing high reproducibility across biological replicates within each treatment group. (**B**) Distribution of sample coefficient of variation (CV) for EGF, vehicle, and VGF-treated samples, with median CV values indicated for each group. (**C**) Boxplots of log_2_ phosphosite intensities before (original) and after normalization (normalized), showing improved consistency across samples. (**D**) Density plots of log_2_ intensities before (normal) and after normalization (corrected), illustrating alignment of signal distributions across treatment groups.
(TIF)

S2 Fig Differential phosphosite analysis of EGF and VGF compared to vehicle. (**A**) Volcano plot showing differential phosphosite abundance between EGF and vehicle-treated HFFs, plotted as log_2_ fold change versus −log_10_(FDR), with significantly regulated phosphosites defined by FDR < 0.05 and |log_2_ fold change| ≥ 1. (**B**) Volcano plot showing differential phosphosite abundance between VGF and vehicle-treated HFFs using the same thresholds. (**C**) Bar plot summarizing significantly regulated phosphosites (FDR < 0.05) in the EGF versus vehicle comparison, grouped by amino acid residue (serine, threonine, tyrosine) and by the condition in which each site shows higher abundance. (**D**) Bar plot summarizing significantly regulated phosphosites (FDR < 0.05) in the VGF versus vehicle comparison, grouped by amino acid residue and direction of change.
(TIF)

S3 Fig Threonine-centered motif analysis and pathway-level phosphorylation differences between EGF and VGF. (**A, B**) Sequence logos of threonine-centered (pT) phosphorylation sites using 15-mer windows for phosphosites enriched in EGF (**A**) or VGF (**B**), showing amino acid frequency patterns surrounding the phosphorylation site. (**C, D**) Kinase motif enrichment analysis of threonine-centered phosphosites, showing enrichment of known kinase recognition motifs (log_2_ odds ratio, foreground vs background) for EGF-enriched (**C**) and VGF-enriched (**D**) sites. (**E, F**) Motif classification of threonine-centered phosphosites into kinase families based on predefined sequence patterns (REGEX), summarizing enrichment across major kinase groups for EGF-enriched (**E**) and VGF-enriched (**F**) sites. (**G**) Heatmap of relative phosphorylation changes (log_2_ fold change) across selected phosphosites within EGFR-related signaling pathways, comparing EGF and VGF treatments across pairwise contrasts. (**H**) Targeted validation of representative signaling pathways downstream of EGFR. HFFs were starved for 48h as described in the methods section and treated with 5 µg/mL VGF or EGF peptide or infected with MOI 2 of WT-VACV or vΔVGF, as indicated. Phosphorylated EGFR (Y1068) and AKT (S473) were examined by Western blot at 4 hours post treatment. Total EGFR and AKT are shown as corresponding protein controls. The blots were from different lanes on the same gel and the dashed lines indicate that some non-relevant lanes were removed (Image representative of n = 3 biological replicates).
(TIF)

S4 Fig RNA-seq workflow and global transcriptional differences between EGF and VGF treatments. (**A**) Experimental workflow for transcriptomic analysis. HFFs were serum starved for 48 h followed by treatment with vehicle, EGF, or VGF for 4 h or 8 h. Total RNA was isolated, libraries were prepared for next-generation RNA sequencing, and reads were mapped for downstream analysis. Transcriptomics was performed with three independent biological replicates (n = 3) per treatment group. (**B, C**) Principal component analysis (PCA) of RNA-seq data at 4 h (**B)** and 8 h (**C**), showing separation of samples by treatment condition. (**D, E**) Volcano plots showing differential gene expression between EGF and VGF-treated samples at 4 h (**D)** and 8 h (**E**), plotted as log_2_ fold change versus −log_10_(adjusted p-value), with significantly regulated genes defined by FDR < 0.05 and |log_2_ fold change| ≥ 1. Together, these analyses demonstrate distinct and time-dependent transcriptional responses between EGF and VGF treatments. Fig S4A *Created in BioRender. Pant, A. (2026)* https://BioRender.com/b7fzkhk.
(TIF)

S5 Fig Phospho-only and RNA-only pathway enrichment distinguishes EGF- and VGF-specific signaling programs. (**A, B**) Reactome pathway enrichment analysis of phosphoproteomics-only (phospho-only) data showing pathways enriched in EGF (**A**) or VGF (**B**) relative to the other condition. Enrichment is based on phosphosite-level statistics mapped to genes, with significance represented as −log_10_(BH-adjusted p-value) and bubble size indicating gene set size. (**C, D**) Reactome pathway enrichment analysis of RNA-seq-only (RNA-only) data showing pathways enriched in EGF (**C**) or VGF (**D**), based on gene-level differential expression analysis. Phospho-only pathways enriched in EGF are primarily associated with RNA processing, clathrin-mediated endocytosis, and Rho-family GTPase signaling, whereas phospho-only pathways enriched in VGF highlight Rho/PAK signaling, growth factor signaling, and regulatory programs linked to TP53 and epigenetic control. In contrast, RNA-only pathways enriched in EGF are dominated by interferon and innate immune signaling programs, while RNA-only pathways enriched in VGF are associated with extracellular matrix organization, DNA replication, and cell-cycle progression.
(TIF)

S1 File Representative phosphosites contributing to EGF- and VGF-associated Hallmark and Gene Ontology Biological Process pathway enrichments. The phosphosites marked with the asterisk (*) signs were identified in both analyses.
(XLSX)

S2 File List of genes included in targeted pathway analysis. (XLSX)
